# Evaluation of low-volume post-dilution online hemodiafiltration with Japanese high-performance hemodiafilters

**DOI:** 10.1007/s10047-020-01167-y

**Published:** 2020-04-07

**Authors:** Kenji Sakurai, Hiromi Hosoya, Yoshitaka Kurihara, Fumi Yamauchi, Ayumi Suzuki, Kaori Kurosawa, Takeshi Saito

**Affiliations:** Hashimoto Clinic, 3-21-5 Hashimoto Midori-ku, Sagamihara, Kanagawa 252-0143 Japan

**Keywords:** Post-dilution online hemodiafiltration, High-volume post-dilution online hemodiafiltration, Low-volume post-dilution online hemodiafiltration, Japanese hemodiafilter, α_1_-Microglobulin

## Abstract

**Purpose:**

To assess the removal performance of low-volume post-hemodiafiltration (HDF) with Japanese hemodiafilters and the removal performance with 20 % reduction in the total dialysate flow rate (*Q*_d_total).

**Methods:**

Subjects were 8 patients undergoing pre-HDF. Study 1: Post-HDF was performed at a blood flow rate (*Q*_b_) of 250 mL/min and a total volume of substitution fluid (Vs) of 12 L/session(s) for 4 hrs using Fineflux-210Seco (FIX), ABH-21PA (ABH), and NVF-21H (NVF). We assessed removal efficiency of small molecular solutes, low-molecular-weight-proteins and the amount of albumin loss. Study 2: Post-HDF was performed at Vs of 12 L/s under G-1, *Q*_d_total of 500 and *Q*_b_ of 250 mL/min; G-2, *Q*_d_total of 400 and *Q*_b_ of 250 mL/min; and G-3, *Q*_d_total of 400 and *Q*_b_ of 300 mL/min. Removal efficiency was compared and analyzed between these conditions.

**Results:**

Study 1: The results using FIX, ABH and NVF are shown in order. The Kt/*V* were 1.8, 1.9 and 1.8. The β_2_-Microglobulin (MG) removal rate (RR) (%) were 81.2, 83.1 and 82.8, and the α_1_-MG RR were 37.4, 40.2 and 38.5, respectively. Study 2: The results in G-1, 2 and 3 are shown in order. The Kt/*V* and the RR of small solutes, were significantly higher in G-3. The β_2_-MG RR (%) were 81.2, 80.1 and 81.0, and the α_1_-MG RR were 37.4, 37.5 and 38.0, respectively.

**Conclusions:**

Low-volume post-HDF performed at *Q*_b_ of 250 mL/min with Japanese high-performance hemodiafilters exhibited favorable removal efficiency for all solutes. Even with 20 % reduction in *Q*_d_total, the removal performance was also favorable.

## Introduction

At the end of 2017, in Japan, there were 334,505 dialysis patients, 21.1 % of whom were receiving online (OL) hemodiafiltration (HDF). In Japan, the pre-dilution OL-HDF (pre-HDF) is used in 84.4 % of patients on OL-HDF. Kikuchi et al. reported that pre-HDF with a volume of substitution fluid (Vs) of 40 L or more favorably affects patient prognosis compared to pre-HDF with Vs of 40 L or less and hemodialysis (HD) [1]. Conversely, in Europe, OL-HDF has been performed in the post-dilution OL-HDF (post-HDF). High-volume post-HDF with a high blood flow rate (*Q*_b_) has been reported to improve patient prognosis, and its effect has been reported to be enhanced with increased Vs [2–4].

In Japan, post-HDF has not been widely accepted, mainly because albumin (Alb) loss was difficult to control in post-HDF and because hemodialysis with high *Q*_b_ was not preferable. We previously reported that pre-HDF was superior to post-HDF in terms of biocompatibility [5]. Later, we reported that post-HDF using recent high-performance hemodiafilter efficiently removed low-molecular-weight protein (LMWP), caused only mild Alb loss, and was comparable with pre-HDF in terms of biocompatibility [6]. Our study showed that high-efficiency post-HDF could be achieved without high *Q*_b_ and high Vs by selecting an appropriate hemodiafilter.

In Study 1 of the present study, post-HDF was performed with 3 types of Japanese high-performance hemodiafilters at Vs of 12 L/session (s) and *Q*_b_ of 250 mL/min, and the removal performance was assessed. Then, we examined whether this low-volume post-HDF with moderate *Q*_b_ exhibited removal efficiency adequate for HDF. In Study 2, removal performance of post-HDF with fixed Vs of 12 L/s and 20 % reduction in the total dialysate flow rate (*Q*_d_total) was assessed to examine whether the dialysate volume could be reduced.

## Patients and methods

### Ethical approval

All subjects enrolled in this research have given their informed consent. The study has been approved by our institutional committee on human and/or animal research, and this protocol has been found acceptable by them (approved number: 2019-05).

### Study design and population

**Fig. 1:**
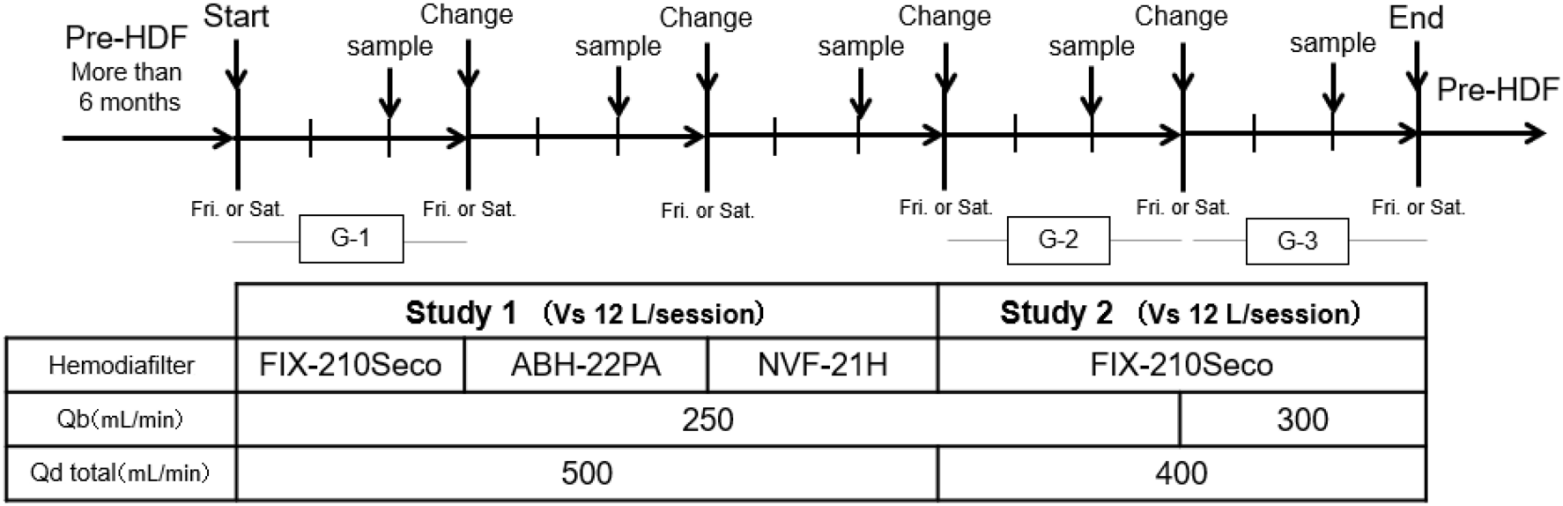
Design of study 1 and study 2

This prospective, single-center study included 8 stable dialysis patients undergoing maintenance dialysis by pre-HDF in our clinic (Table 1). Each patient had received pre-HDF 3 times per week (i.e., on Monday, Wednesday, and Friday or on Tuesday, Thursday, and Saturday) for 6 months or longer (Fig. 1).

**Table 1:** Patient background characteristics

Patient number	Sex	Age (year)	Dialysis vintage (months)	Cause of ESRD	Dry weight (kg)
1	M	44	305	VUR	53.4
2	M	75	81	PCK	58.5
3	M	37	36	DMN	64.3
4	M	70	72	DMN	60.5
5	F	44	13	DMN	58.5
6	F	74	163	CGN	41.9
7	F	63	13	Unknown	54.2
8	F	60	238	CGN	44.4
Average		58.4 ± 14.9	115.1 ± 109.3		54.5 ± 7.8

*M* male, *F* female, *ESRD* end-stage renal disease, *VUR* vesicoureteral reflux, *PCK* polycystic kidney disease, *DMN* diabetic nephropathy, *CGN* chronic glomerulonephritis

*Study 1* In the patients, pre-HDF was switched to post-HDF on Friday or Saturday, and post-HDF was performed 3 times under the same conditions. In the third session (on the middle day of the week), samples were collected. According to this dialysis schedule, post-HDF was repeated using 3 types of hemodiafilters for 3 weeks. The hemodiafilters used were Fineflux-210Seco (FIX) (asymmetric triacetate [ATA] membrane, Nipro Co., Osaka, Japan) in the first week, ABH-21PA (ABH) (polysulfone [PS] membrane, Asahi Kasei Medical Co., Ltd. Tokyo, Japan) in the second week, and NVF-21H (NVF) (PS membrane, Toray Medical Co., Ltd., Tokyo, Japan) in the third week (Table 2). Post-HDF was performed at *Q*_b_ of 250 mL/min and Vs of 12 L/s for 4 hrs (Table 3).

**Table 2:** Hemodiafilter specifications

	FIX-210S	ABH-22PA	NVF-21H
Membrane material	ATA	PS	PS
Sieving coefficient β2-microglobulin	0.93	0.78	–
Sieving coefficient albumin	0.01	0.01↓	0.009
Membrane surface area (m^2^)	2.1	2.2	2.1

*ATA* asymmetric triacetate, *PS* polysulfone

**Table 3:** Treatment modes in study 1

	FIX-210S	ABH-22PA	NVF-21H
*Q*_b_ (mL/min)	250		
*Q*_d_ (mL/min)	500		
Treatment time (h)	4		
Substitution fluid			
(L/session)	12		
(mL/min)	50		
Net dialysate flow rate (mL/min)	450		
*Q*_S_ + *Q*_F_ (mL/min)	63.3 ± 5.6	62.6 ± 4.8	62.2 ± 5.0
Filtration fraction (%)			
Blood flow	25.3 ± 2.2	25.0 ± 1.9	24.9 ± 2.0
Plasma flow	41.3 ± 3.6	40.8 ± 1.9	41.9 ± 3.9

*Q*_*b*_ blood flow rate, *Q*_*d*_ dialysate flow rate

*Study 2* The patients who participated in Study 2 were the same as those who participated in Study 1. Post-HDF using FIX was performed at a fixed Vs of 12 L/s, *Q*_d_ reduced to 400 mL/min, and *Q*_b_ of 250 mL/min in Group 2 (G-2) or 300 mL/min in Group 3 (G-3). The removal efficiency in these groups was compared with that of post-HDF performed at total *Q*_d_ (*Q*_d_total) of 500 mL/min and *Q*_b_ of 250 mL/min in Group 1 (G-1: this was done in the first term of study 1). In Study 2, post-HDF was also performed 3 times under the same conditions, and samples were collected in the third session (Table 4).

**Table 4:** Treatment modes in study 2

	G-1	G-2	G-3
*Q*_b_ (mL/min)	250	250	300
*Q*_d_ (mL/min)	500	400	400
Treatment time (h)		4	
Substitution fluid			
(L/session)		12	
(mL/min)		50	
Net dialysate flow rate (mL/min)	450	350	350
*Q*_S_ + *Q*_F_ (mL/min)	63.3 ± 5.6	63.3 ± 5.1	62.9 ± 5.0
Filtration fraction (%)			
Blood flow	25.3 ± 2.2	25.3 ± 2.0	21.0 ± 1.7^#,++^
Plasma Flow	41.3 ± 3.6	42.9 ± 4.2	35.3 ± 3.2^#,++^

*Q*_*b*_ blood flow rate, *Q*_*d*_ dialysate flow rate, *vs*. versus, *G-1* group 1, *G-2* group 2, *G-3* group 3

Friedman test, ^#^*p* < 0.05 vs. G-1, ^++^*p* < 0.01 vs. G-2

### Data collection

To assess removal performance, we measured the standardized dialysis dose (Kt/*V*); the removal rates and amounts of urea [molecular weight (MW): 60 Da], creatinine (MW: 113 Da), phosphorus (MW: 30.97 Da), ß_2_-microglobulin (MG) (MW: 11.8 kDa), and α_1_-MG (MW: 33 kDa); and the removal rate of prolactin (MW: 22 kDa). To measure the removal amount of each solute and the amount of Alb loss, spent dialysate was pooled at 2 L/h. At the end of a post-HDF session, the pooled fluid was thoroughly stirred, and then samples were collected.

Filtration fractions for post-HDF with blood flow and plasma flow were calculated with the equations presented as follows [7]:

1 $${\text{Filtration}}\,{\text{fraction}}_{{{ }\left( {{\text{Blood}}\,{\text{flow}}} \right)}} = \frac{{Q_{{\text{S}}} + Q_{{\text{F}}} }}{{Q_{{\text{B}}} }} \times 100,$$

2 $$Q_{{\text{p}}} = \left( {1 - \frac{{{\text{Ht}}}}{100}} \right) \times \left( {1 - 0.0107 \times {\text{TP}}} \right) \times Q_{{\text{B}}} ,$$

3 $${\text{Filtration}}\,{\text{fraction}}_{{{ }\left( {{\text{Plasma}}\,{\text{flow}}} \right)}} = \frac{{Q_{{\text{S}}} + Q_{{\text{F}}} }}{{Q_{{\text{p}}} }} \times 100.$$

Of the laboratory data, those affected by concentration due to ultrafiltration after dialysis were corrected for the hematocrit values.

### Statistical analysis

All results are expressed as a mean and standard deviation. The Friedman test was performed to analyze all parameters using StatMate III for Windows (ATMS Corp., Tokyo, Japan). A *p* value less than 5 % was considered as the significance level.

## Results

*Study 1* The respective results are presented in the order of FIX, ABH, and NVF. Kt/*V* values were 1.82 ± 0.29, 1.89 ± 0.32, and 1.83 ± 0.31. There were significant differences between FIX and ABH, and between ABH and NVF in Kt/*V*. The removal rates of urea (%) were 76.9 ± 4.8, 78.3 ± 4.9, and 77.3 ± 5.1, with a significant difference between ABH and NVF. Removal rates of ß_2_-MG (%) were 81.2 ± 2.6, 83.1 ± 4.3, and 82.8 ± 3.7. Removal rates of prolactin (%) were 80.2 ± 3.2, 78.0 ± 9.9 and 77.9 ± 7.3, and removal rates of α_1_-MG (%) were 37.4 ± 3.9, 40.2 ± 8.2 and 38.5 ± 7.0, respectively. Amounts of Alb loss (g/s) were 3.4 ± 0.7, 5.4 ± 2.1, and 5.4 ± 2.8, but no significant difference was observed. Ratios of α_1_-MG/Alb (mg/g) were 44.4 ± 9.3, 33.0 ± 9.7, and 36.9 ± 10.5. For 1 g of Alb loss, the removal amount of α_1_-MG was higher for FIX, but it did not significantly differ between the hemodiafilters (Table 5). During post-HDF, the increase in transmembrane pressure (TMP) was mild for all hemodiafilters, and it remained at 100 mmHg or lower until the end of each session.

**Table 5:** Results of study 1

	FIX-210S	ABH-22PA	NVF-21H
Kt/*V*	1.82 ± 0.29	1.89 ± 0.32	1.83 ± 0.31^+^
Removal rate (%)			
Urea	76.9 ± 4.8	78.3 ± 4.9**	77.3 ± 5.1
Creatinine	70.9 ± 5.3	71.9 ± 4.8	71.3 ± 5.5
Phosphorus	62.3 ± 5.3	66.2 ± 4.7	63.3 ± 7.4
β_2_-microglobulin	81.2 ± 2.6	83.1 ± 4.3	82.8 ± 3.7
Prolactin	80.2 ± 3.2	78.0 ± 9.9	77.9 ± 7.3
α_1_-microglobulin	37.4 ± 3.9	40.2 ± 8.2	38.5 ± 7.0
Removal amount (g)			
Urea	14.2 ± 4.2	12.0 ± 3.5	11.6 ± 2.8
Creatinine	1.9 ± 0.4	1.8 ± 0.4	1.8 ± 0.4
Phosphorus	1.1 ± 0.2	1.1 ± 0.2	1.1 ± 0.2
β_2_-microglobulin	0.222 ± 0.026	0.199 ± 0.025**	0.198 ± 0.027^#^
α_1_-microglobulin	0.146 ± 0.030	0.163 ± 0.026	0.180 ± 0.054
Loss of albumin in dialysate (g)	3.4 ± 0.7	5.4 ± 2.1	5.4 ± 2.8
Serum Albumin level (g/dL)	3.73 ± 0.14	3.64 ± 0.11	3.66 ± 0.18
α_1_-microglobulin/albumin	44.4 ± 9.3	33.0 ± 9.7	36.9 ± 10.5

*Vs*. versus

Friedman test, **p < 0.01 vs. FIX-210S, ^#^*p* < 0.05 vs. FIX-210S, ^+^*p* < 0.05 vs. ABH-22PA

*Study 2* The respective results are presented in the order of G-1, G-2, and G-3. Kt/*V* values were 1.82 ± 0.29, 1.77 ± 0.28, and 2.02 ± 0.36. As with the removal rates of urea and creatinine, Kt/*V* was significantly highest in G-3. Removal rates of ß_2_-MG (%) were 81.2 ± 2.6, 80.1 ± 4.2, and 81.0 ± 3.6; the rate in G-1 was significantly higher than that in G-2 (*p* < 0.05). Removal rates of prolactin (%) were 80.2 ± 3.2, 79.6 ± 5.1, and 80.5 ± 5.0. Removal rates of α_1_-MG (%) were 37.4 ± 3.9, 37.5 ± 6.5, and 38.0 ± 5.8. Amounts of Alb loss (g/s) were 3.4 ± 0.7, 3.8 ± 1.1, and 4.0 ± 0.8, showing no significant difference. Removal amounts of ß_2_-MG (g) were 0.222 ± 0.03, 0.199 ± 0.03, and 0.198 ± 0.03; the amount was significantly highest in G-1. Removal amounts of α_1_-MG (g) were 0.146 ± 0.033, 0.163 ± 0.03, and 0.180 ± 0.005; the amount was significantly highest in G-3. In addition, ratios of α_1_-MG/Alb were 44.4 ± 9.3, 40.2 ± 9.6, and 42.0 ± 8.2 (Table 6).

**Table 6:** Results of study 2

	G-1	G-2	G-3
Kt/*V*	1.82 ± 0.29	1.77 ± 0.28	2.02 ± 0.36^#,++^
Removal rate (%)			
Urea	76.9 ± 4.8	76.1 ± 4.8	80.2 ± 5.0^#, ++^
Creatinine	70.9 ± 5.3	69.8 ± 4.7	74.6 ± 5.2^#, ++^
Phosphorus	62.3 ± 5.3	60.9 ± 11.9	66.5 ± 7.7
β_2_-microglobulin	81.2 ± 2.6	80.1 ± 4.2*	81.0 ± 3.6
Prolactin	80.2 ± 3.2	79.6 ± 5.1	80.5 ± 5.0
α_1_-microglobulin	37.4 ± 3.9	37.5 ± 6.5	38.0 ± 5.8
Removal amount (g)			
Urea	14.2 ± 4.2	11.8 ± 3.0	12.9 ± 2.9
Creatinine	1.9 ± 0.4	1.8 ± 0.4	1.9 ± 0.4
Phosphorus	1.1 ± 0.2	1.1 ± 0.2	1.2 ± 0.1
β_2_-microglobulin	0.222 ± 0.026	0.202 ± 0.024*	0.202 ± 0.023^##^
α_1_-microglobulin	0.146 ± 0.030	0.145 ± 0.032	0.162 ± 0.026^##,+^
Loss of albumin in dialysate (g)	3.4 ± 0.7	3.8 ± 1.1	4.0 ± 0.8
Serum Albumin level (g/dL)	3.73 ± 0.14	3.69 ± 0.25	3.61 ± 0.20
α_1_-microglobulin/albumin	44.4 ± 9.3	40.2 ± 9.6	42.0 ± 8.2

*Vs.* versus, *G-1* group 1, *G-2* group 2, *G-3* group 3

Friedman test, *p < 0.05 vs. G-1, ^#^*p* < 0.05 vs. G-1, ^##^*p* < 0.01 vs. G-1, ^+^*p* < 0.05 vs. G-2, ^++^*p* < 0.01 vs. G-2

## Discussion

The FIX series consists of 4 types of products (i.e., FIX-U, -S, -E, and -M), and the NVF series consists of 3 types of products (i.e., NVF-P, -H, and -M). The membranes of the FIX-U and NVF-P have the largest pore size among the respective series. From these products, we selected and used the FIX-S and NVF-H, with which post-HDF was assumed to yield adequate removal performance for LMWP and to keep Alb loss within the adequate range. As for ABH-PA, which is the only available type of the product, there was no alternative.

In the present study, because post-HDF was performed at *Q*_d_total of 500 mL/min and Vs of 12 L/s, the net *Q*_d_ was 450 mL/min. Under this condition, the efficiency in removing small molecular solutes by diffusion was expressed as Kt/*V* of 1.8 or higher and removal rates of 75 % or higher for urea and 70 % for creatinine, which were favorable. We previously reported that HDF should be set with target removal rates of 80 % for ß_2_-MG and 35 % for α_1_-MG when dialysis patients with various complications are treated [8, 9]. Post-HDF performed under the conditions set in the present study was a therapeutic strategy demonstrating the original features of HDF (i.e., efficient removal of middle- to large-molecular-weight solutes), because the removal rates were 80 % or higher for ß_2_-MG and 35 % or higher for α_1_-MG.

The European style of post-HDF requires a high Vs (15–30 L/s) to improve efficiency in removing solutes by convection [10]. High Vs inevitably leads to high *Q*_b_ to prevent hemoconcentration. For example, in the ESHOL study, *Q*_b_ and Vs were reported to be 384–392 mL/min and 20.8–21.8 L/s, respectively [3]. There is no other way but to state that high Vs and high *Q*_b_ are required, because the removal performance of hemodiafilters used for HDF in Europe is unsatisfactory for substances in the LMWP range.

The European style of post-HDF at high *Q*_b_ is associated with 2 disadvantages. The first disadvantage is the risk of excessive loss of amino acids. Because amino acids are small molecular solutes, their loss increases with higher *Q*_b_. Excessive loss of amino acids obviously has an adverse effect on the nutritional status of patients. The second disadvantage is the increased risk of the development of micro air-bubbles in the blood tube circuit. Stegmayr et al. reported that when *Q*_b_ is 300 mL/min or higher, micro air-bubbles always develop in the blood tube circuit, enter the body via a dialysis membrane, and adversely affect the body [11–13]. With *Q*_b_ of 250 mL/min, at which post-HDF was performed in the present study, the risk of the development of micro air-bubbles is low. Based on these 2 disadvantages, the Japanese style of post-HDF, characterized by low-volume of substitution fluid and moderate *Q*_b_ using Japanese high-performance hemodiafilters, clearly appears to be superior to the European style of post-HDF. Furthermore, the former may also be superior in biocompatibility, because the increase in TMP during post-HDF is mild [14].

Among the 3 types of hemodiafilters used in the present study, Kt/*V*, removal rate of urea, removal rate and amount of β_2_-MG showed significant differences in several comparisons. However, it is unlikely that these differences could result in a problem in clinical practice. There was no significant difference in the amount of Alb loss. When using FIX, however, even though the serum level of Alb was high, the amount of Alb loss was low and its standard deviation was also small. The ratio of α_1_-MG/Alb was also at its highest when FIX was used (Table 5). These results indicate that FIX is capable of suppressing Alb loss and efficiently removing α_1_-MG to some extent. These findings imply that the radii of pores on the FIX vary within a small range, and that there are none or only a few shunt pores (i.e., non-standard, large pores). Because the problem with conventional post-HDF is the difficulty in controlling Alb loss, under the present circumstances, the FIX appears to be the most appropriate hemodiafilter for post-HDF among those 3 types of hemodiafilters.

When post-HDF was performed at Vs of 12 L/s for 4 hrs with 20 % reduction in *Q*_d_total (i.e., *Q*_d_total of 400 mL/min), the net *Q*_d_ was 350 mL/min, and the removal efficiency for small molecular solutes was only slightly decreased. Furthermore, because Vs remained the same, it is not surprising that the removal efficiency for LMWP was unchanged. When *Q*_b_ was 300 mL/min, the removal efficiency for small molecular solutes was significantly improved. However, it should be kept in mind that the loss of amino acids and the risk of development of micro-air-bubbles increase with higher *Q*_b_.

## Conclusions

Low-volume post-HDF with moderate *Q*_b_ using Japanese high-performance hemodiafilters can efficiently remove small-, middle-, and large-molecular-weight solutes. In addition, even when *Q*_d_total is reduced by 20 %, favorable removal performance is achieved for all solutes. Thus, post-HDF will be presumably performed more often, and the European style of high-volume post-HDF is considered unnecessary in Japan.
